# The Two Dimensions of Nutrition for the Planet: Environment and Health

**DOI:** 10.1007/s13668-025-00642-3

**Published:** 2025-03-20

**Authors:** Gökçe Sueda Aydoğdu, Makbule Gezmen Karadağ

**Affiliations:** 1 Faculty of Health Sciences, Department of Nutrition and Dietetics, Anadolu University, Eskişehir, Turkey; 2 Faculty of Health Sciences, Department of Nutrition and Dietetics, Gazi University, Ankara, Turkey

**Keywords:** Planetary health, Sustainable diets, Mediterranean diet, New Nordic diet, Carbon footprint

## Abstract

**Purpose of Review:**

Protecting the planet is protecting the future. Food production systems are among the most important human activities threatening planetary health. Diet, food systems, the environment, and health are interconnected. Accordingly, this review aims to assess the effects of nutrition on the planet and the relationship between some types of diets defined as sustainable and the planet and human health.

**Recent Findings:**

Many diets have been proposed to protect the planet and human health, but there is no consensus on which diet is best. It should not be forgotten that planetary health diets, plant-based diets, and vegetarian/vegan diets can reduce environmental pressure. Still, they cannot have the same effect in every country, and these diets may have different effects depending on the differences in the countries' income level, nutritional culture, and food systems. Moreover, it should not be overlooked that these diets may cause difficulties in terms of adaptation, cause deficiencies in some nutrients, and may not be suitable for all segments of society. Sustainable diets such as the Mediterranean and New Nordic, as well as Dietary Approaches to Stop Hypertension, are more flexible and acceptable.

**Summary:**

Instead of a globally recommended reference diet to protect the planet and human health, each country can analyze its food systems and choose the most appropriate food production methods and sustainable diet style to reduce environmental burden, improve health, and create policies accordingly, which can help achieve sustainable goals faster.

## Introduction

Food, environment, and health are interrelated and cannot be considered independently. People's dietary patterns affect the environment, and the environment also affects the types of diets people prefer [1]. In addition, the foods and beverages consumed impact health [1], and individual dietary preferences influence food systems. Food production systems are among the most important causes of environmental changes, pushing the planet's boundaries and posing a risk to the planet [2] and human health [3]. In particular, food production systems can cause environmental pressures with effects such as increasing greenhouse gas (GHG) emissions, biodiversity loss, land use, and water use [4]. Due to the increasing population and the advancing process, there is expected to be an increase of 60% in food demand by 2050. This situation may raise the environmental pressure on food systems, causing the depletion of natural resources and leading to results such as being unable to reach enough food and not achieving food safety. As a result, human health is at risk [5].

The EAT-Lancent Commission has recently emphasized the importance of win–win diets that reduce the adverse effects of food systems and protect the planet and human health. They have created a global reference diet for this purpose [6]. Win–win diets are diets that are both environmentally sustainable and have positive impacts on health [6]. In this context, the concept of a sustainable diet gains importance. Sustainable diets support the health and well-being of people in all dimensions, have low environmental pressure and effect, have a safe, accessible, affordable, and fair distribution, and are culturally acceptable [7]. These diets aim to protect and advance people's health, prevent biodiversity loss, and protect the planet [7]. However, it remains unclear which, if any, is universally best for both planetary and human health. This review aims to assess the effects of nutrition on the planet and the relationship between some types of diets defined as sustainable for the planet and human health.

## Method

Pubmed, Science Direct, and Google Scholar databases were searched for this narrative review. The literature review was conducted between May 30 and June 30, 2023. Between June 30 and July 30, 2024, the search was updated. This narrative review mainly focuses on studies published after 2014. 90% of the studies included in the review are from 2014 and later. Only studies published in English were included. The words planetary health, greenhouse gas emissions, nitrogen cycle, phosphorus cycle, freshwater use, land use, biodiversity loss, environmental footprint, carbon footprint, water footprint, sustainable diets, plant-based diets, vegetarian diets, Mediterranean diet, New Nordic diet, Dietary Approaches to Stop Hypertension, health, cardiovascular disease, diabetes, kidney disease, obesity were used alone or in combination. Modeling studies, cross-sectional studies, cohort studies, human intervention studies, and systematic/meta-analyses were included. Reference lists of the studies found were also utilized. Systematic/meta-analysis studies were prioritized, especially studies conducted in health.

## Planetary Health and Food Systems

The Anthropocene epoch is the epoch in which human activities are the primary reason for changes in world systems [6]. The influence of human actions on Earth's systems has risen in the last ten years and has caused the deterioration of most natural systems [8]. In particular, human actions have caused greenhouse gases such as carbon dioxide, nitrous oxide, and methane in the atmosphere to increase, which has increased the temperature of the world and led to global warming. Global warming has led to such distortions as a decrease in freshwater resources, desertification, a decrease in agricultural productivity, ecosystem degradation, ocean acidification, biodiversity loss, and stratospheric ozone depletion [9]. These deteriorations threaten both the planet and human health [8].

The notion of planetary health is the idea that human health and human civilization rely on the natural systems on Earth and the intelligent management of these natural systems [10]. These natural systems are rapidly deteriorating due to human activities [8]. On the other hand, the concept of planetary boundary aims to describe the environmental boundaries within which humankind can safely operate in order not to damage the planet [11]. There are nine planetary boundaries. These are climate change, atmospheric aerosol load, stratospheric ozone depletion, biogeochemical cycles (phosphorus and nitrogen cycles), ocean acidification, land use change, biosphere integrity (biodiversity loss), freshwater use, and presentation of novel entities [11]. Exceeding planetary boundaries threatens human health [3].

Food systems are one of the main driving forces of the Earth's system pushing planetary boundaries [2]. Food systems consist of activities and outputs that cover all stages and elements (inputs, people, institutions, infrastructure, environment, etc.) associated with the manufacture, processing, distribution, preparation, and consumption of food [12] and have human, environmental, and socioeconomic dimensions [13]. Food systems and the environment are in a two-way interaction. Environmental inputs such as soil, water, and climate affect food systems. At the same time, food systems also have environmental effects such as depletion of natural resources, air pollution, GHG production, deforestation, eutrophication, rising temperatures, and rising sea levels [14]. The fact that food systems cannot provide adequate nutrition to the growing population cannot distribute nutrients fairly and evenly (excessive food consumption, insufficient food consumption), and damage the environment and natural resources proves that they cannot fulfill their duties as they should [15].

**Fig. 1:**
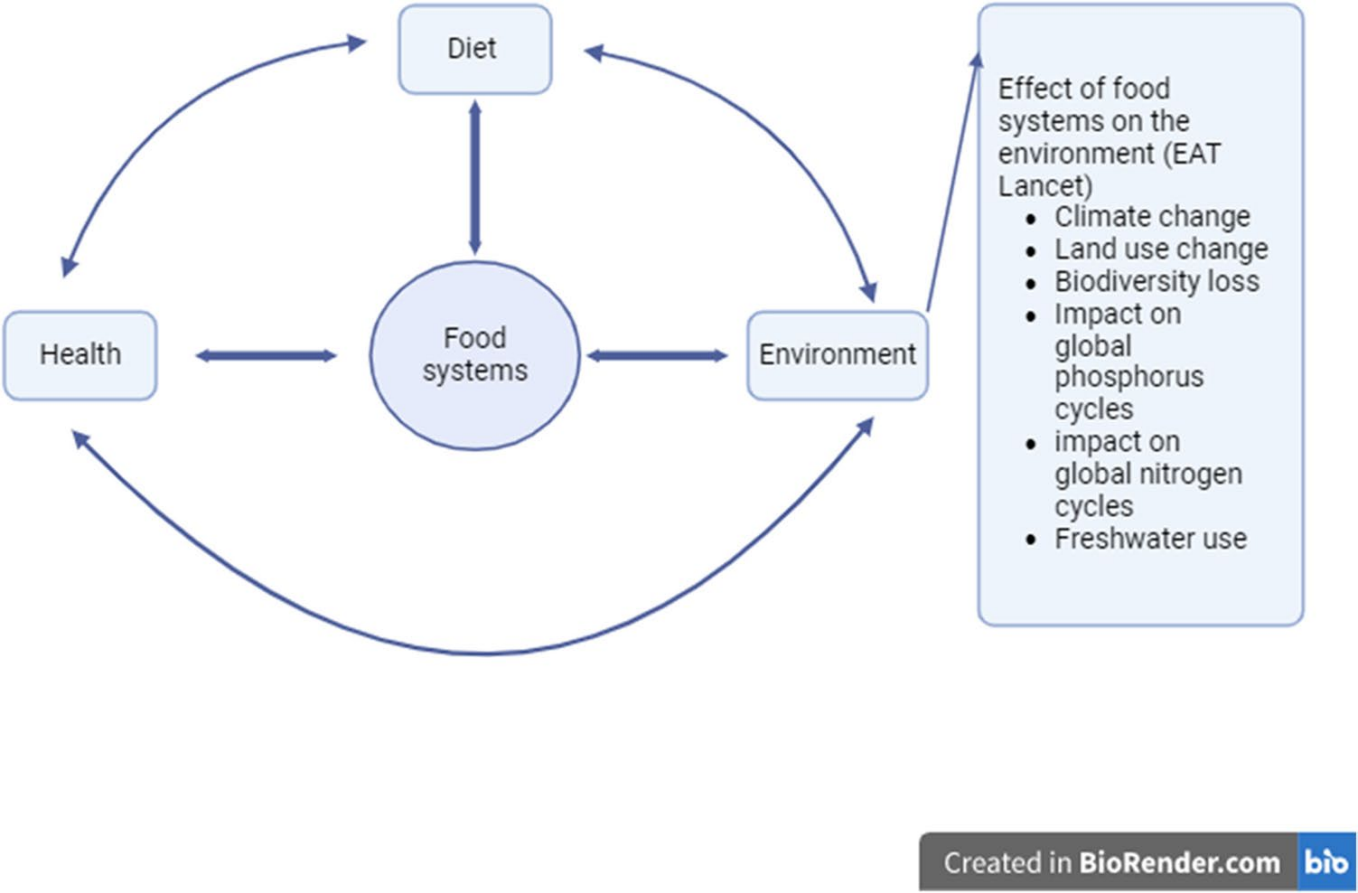
Food systems, health, diet, and environment interactions

The communication between environment, health, food systems, diet, and the environmental impact of food results according to the EAT Lancet are given in Fig. 1 [6].

Food production systems are related to about 20–35% of worldwide GHG emissions [7], 80% of biodiversity loss, 70% of water withdrawal for irrigation in agriculture, and 40% of land use [4]. The kinds of diets that people prefer and food systems are closely connected [16]. In addition, diets establish the connection between the environment and human health [17] and have important effects on the environment [4].

While climate is an input for food systems, food systems are also an important factor affecting the climate [14]. The primary reason for climate change is GHG emissions [18]. Food production makes up about 30% of worldwide GHG emissions [19]. Agricultural production alone accounts for 10–12% of GHG production; four-fifths of agricultural emissions originate from the livestock sector [20]. Another important part of food systems is water [21]. 70% of all freshwater resources are utilized for agriculture [22]. With these water sources, about 20% of the cropland is irrigated, which accounts for 40% of global food production [23]. It is required in pre-production (e.g., production of farm entries such as seeds, fertilizer, and energy), post-production (e.g., carrying and distribution), food preparation and consumption (e.g., washing and cooking), and waste processes [24]. Food systems must be improved to meet the sustainable development goals for clean water and sanitation [21]. In order to increase the efficiency of the foods produced in agriculture, fertilizers containing nitrogen and phosphorus are applied as standard [11]. Food systems are responsible for the anthropogenic deterioration of the nitrogen cycle by producing surplus fixed nitrogen. Excessively constant nitrogen increases the greenhouse impact, depletes stratospheric ozone, acidifies rainwater, causes eutrophication in bays and estuaries, pollutes drinking water, and pressures ecosystems [25]. Phosphorus is a significant input for agricultural food production. The restricted availability of phosphorus causes excellent interest in phosphorus for food security worldwide [26]. Phosphorus used in food systems harms the environment beyond acceptable limits by causing the deterioration of the phosphorus balance in the world [27]. It is hazardous because it leads to the eutrophication of wetlands [26].

As there may be many reasons for land system changes, increasing demand for soil-based food production is among the most important. The transformation of natural ecosystems to agricultural land causes a quarter of the greenhouse gases emitted from food systems [28]. One of the most considerable causes of deforestation on the Earth is food systems, responsible for about 80% of this loss [29].

Food systems are among the most significant reasons for biodiversity loss. In the last 50 years, the transformation of natural ecosystems for food production or grassland, the inputs used during food production, such as fertilizers, pesticides, etc., have been the main reason for habitat loss and, accordingly, reduced biodiversity [30].

Food systems put the continuity of natural resources at risk; in other words, they cause pressure on natural resources. This situation negatively affects the sustainable nutrition process. In order to achieve sustainable development goals, minimizing the adverse effects of food systems and maintaining sustainable nutrition is a global problem [31]. This requires a sustainable transformation of food systems [32]. Many different practices can be implemented to ensure the sustainability of food systems at different stages [14], such as choosing more sustainable methods in agriculture [33], minimizing food waste at each stage, and reducing plastic use [14]. Ensuring the sustainability of food systems is a significant aspect of reducing the environmental impact of foods and diets [14]. Determining the environmental impact of food systems is important for sustainability. One of the ways to estimate the environmental impacts of food systems is environmental footprints [31]. Many environmental footprints are associated with food systems, such as carbon, water, nitrogen, and phosphorus. It has been suggested that 10 of the 15 footprints associated with food systems are related to agriculture [34]. An increase in the environmental footprint of a food or diet indicates an increase in the environmental pressure of that food. For example, an increase in carbon footprint reflects an increase in greenhouse gas emissions [31], while an increase in water footprint reflects an increase in water use [35]. Table 1 shows the different types of studies evaluating the environmental impacts of some diets that are considered sustainable.

**Table 1:** Different Types of Studies Assessing the Environmental Impacts of Some Sustainable Diets

Author, year	Study design	Place of the study / Sample of the study	Diet evaluated/index used to evaluate diet/dietary intervention implemented	Environmental footprint assessed, Effect of diet on the environment	Main results
Germani et al. 2014 [36]	Dietary pattern assessment	Italy	- Current Italian diet (7-day food consumption of the Italian population) -Mediterranean diet (MD)	-Carbon footprints -Water footprints -Ecological footprints	MD Carbon footprints: 17,04 CO_2_equ/kg/week Water footprints:13781 L/kg/week Ecological footprints: 129 m^2^/kg/week Current Italian diet Carbon footprints: 24,09 CO2equ/kg/week Water footprints:16745 L/kg/week Ecological footprints: 17 m^2^/kg/week
Castañé et al. 2017 [37]	Dietary pattern assessment	Spain	-A weekly menu suitable for the MD -A weekly menu suitable for a vegan diet	-İmpact categories of global warming potential (GWP) -Regional biodiversity impact (RBI)	MD GWP:20 kg CO2equ//person x week RBI: 2,2 × 10 ^−8^ potential species loss/person week Vegan diet GWP: ~ 12 kg CO2equ//person x week RBI: 6,1 × 10^−9^ potential species loss/person week
Rosi et al. 2017 [38]	Cross-sectional	Italy −51 omnivores, −51 ovo-lacto-vegetarians, −51 vegans	-Seven-day food consumption record -Italian Mediterranean Index	-Carbon footprints -Water footprints -Ecological footprints	The diet of omnivores compared to ovo-lacto vegetarians and vegans Carbon footprints↑ Water footprints↑ Ecological footprints↑ There is no difference in environmental footprint between ovo-lacto vegetarians and vegans
Ulaszewska et al. 2017 [39]	Dietary pattern assessment	Italy	-MD - New Nordic diet	-GHG emission	MD GHG emission: 23.56 kg CO2 eq/week New Nordic diet GHG emission: 25.8 kg CO2 eq/week
Springmann et al. 2018 [40]	Global modeling	More than 150 countries	-Scenario: Replacement of 25–100% of animal-based foods with plant-based foods	-GHG emission -Cropland use -Freshwater use -Nitrogen application -Phosphorus application	Scenario: -GHG: 20%↓−84%↓ (particularly effective in high-İncome countries) -Freshwater use: 4%↑−16%↑ -Cropland use: 12%↓ (in upper-middle-income) 29%↓ (in high-income countries) 1%↑ (in lower-middle-income countries) 15%↑(in low-income countries) -Nitrogen application: ↓22% (in upper-middle-income) ↓38% (in high-income countries) 7%↑ (in lower-middle-income countries) 1%↑ (in low-income countries) -Phosphorus application:↓25% in upper-middle-income) ↓35%(in high-income countries) 7%↑ (in lower-middle-income countries) 3%↑ (in low-income countries)
Wood et al. 2019 [41]	Diet scenario study-Modeling	––	-Current American diet -A healthy diet -MD -Vegetarian diet -Modeling the transition from the current American diet to other diets -Different daily energy intakes were evaluated for each diet (2000 kcal/day, 2600 kcal/day, 3200 kcal/day)	-Land use -Water footprints -GHG emission -Phosphorus use -Nitrogen use -Energy use	Transition from the current American diet to other diets Land use ↓ (10,8%−71,1%) Water footprints↑ (33%−92%) GHG↓↑ (switching to a vegetarian diet↓) Phosphorus use ↓ (38–43%) Nitrogen use ↓ (38–43%) Energy use ↓↑ (↑ in energy use, excluding vegetarian diets)
Semba et al. 2020 [42]	Modeling	151 countries	-Switching to the EAT-lancet diet	-GHG emission	If all countries adopt the EAT-Lancet diet, a GHG of 23% ↓ GHG in 101 countries ↓ GHG in 36 countries 12–283% ↑
Belgacem et al. 2021[43]	Diet scenario study	––	-European diet -Western-style diet -MD	-Land use -Water use - Eutrophication -GHG emission	The transition from the European diet to MD Land use↓ Water use↓ Eutrophication↓ GHG↓ The transition from Western diet to MD Land use↓ Water use↓ Eutrophication↓ GHG↓
Cambeses –Franco et al. 2021 [44]	Dietary pattern assessment study	–-	-New Nordic diet -MD	-Carbon footprint -Water footprint	The new Nordic diet Carbon footprint: 3.58 kg CO_2_-person^−1^-day^−1^ Water footprint: 3528 L-person^−1^-day^−1^ New Nordic diet environmental footprint > MD environmental footprint
Tepper et al. 2022 [45]	Cross-sectional	Israel Adult individual (n = 525)	-Adherence to the MD -Adherence to the EAT-Lancet reference diet -Adherence to a sustainable healthy diet	-Land footprint -Water footprint - GHG emission	Compliance with diets↑ Land footprint ↓ GHG↓ Water footprint ↑
Montejano Vallejo et al. 2022 [46]	Cohort	Germany (DONALD study) Individuals aged 15 and over (n = 298)	-EAT-Lancet diet	-GHG emission -Land use	EAT-Lancet diet compliance↑ Land use ↓ GHG↓
Ye Y-X et al. 2023[47]	Cohort	China (n = 57,078)	-Planteray health diet	-GHG emission -Water footprints -Land use footprint	Commitment to a planetary health diet ↑ GHG↓ Water footprints ↑ Land use footprint ↑
García et al. 2023[48]	Cross-sectional	Spain Older adults (n = 6646)	-Adapting to a reduced energy MD	-CO_2_ emissions	Diet adherence↑ CO_2_ emissions↓
Álvarez-Álvarez et al. 2024 [49]	Intervention study	Spain PREDIMED-Plus (n = 5800)	-MD promotion (for one year)	-GHG emission -Eutrophication -Acidification -Energy use -Land use	After one year Adherence to MD↑ Eutrophication↓ Acidification↓ Land use↓
Pınarlı Falakacılar et al. 2024 [50]	Intervention study	Türkiye University students (n = 160)	-Sustainable nutrition course (1 h for 6 weeks)	-Carbon footprint -Water footprint	After a sustained course of intervention Carbon footprint 22% ↓ Water footprint 10% ↓

n: sample size, ↑:increased, ↓:decreased ↔ :unchanged, –-:no information, GHG: greenhouse gas emissions, CO_2:_ Carbon dioxide, Mediterranean diet: MD

## Nutrition, Sustainable Diets and Planet Health

Diets and food systems are closely linked. Diet is an individual's consumption of foods by choosing among the foods produced by the food system. Therefore, the dietary orientation of individuals is the most important reason for the demand for food that drives food systems. Both diet choices and food systems affect each other in two ways [16]. Sustainable healthy diets support the health and well-being of individuals in all dimensions, have low environmental pressure and effect, and are accessible, affordable, safe, equitable, and culturally acceptable diets [7]. The main goals of sustainable diets are to optimize individuals' health and support the protection of biodiversity and the planet [7].

**Fig. 2:**
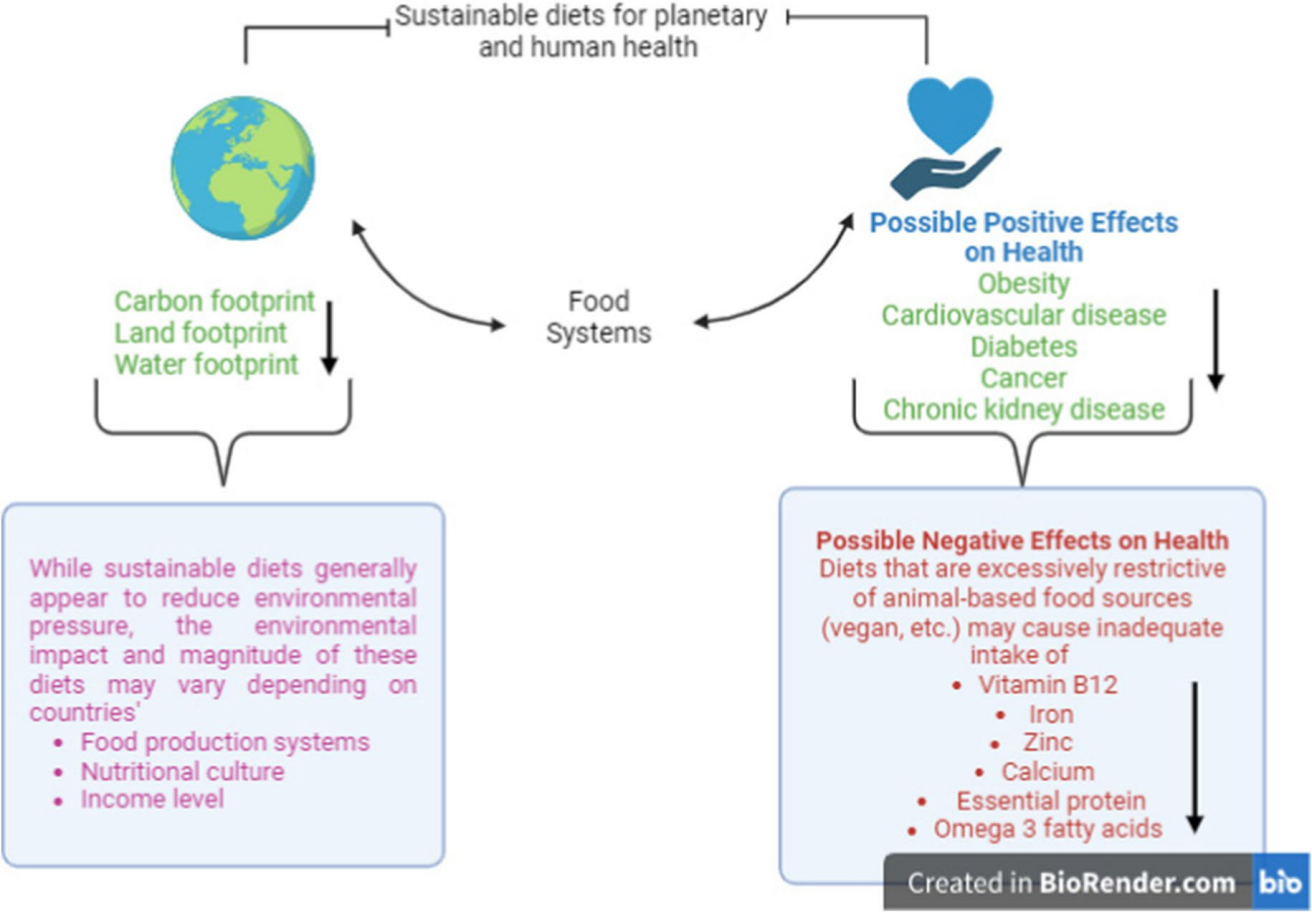
The Potential Impact of Sustainable Diets on Planet and Human Health. The ↓ symbol highlights that sustainable diets reduce some environmental footprints (the most commonly associated footprints are included) and disease risk and that diets that restrict excessive animal-sourced food intake may lead to inadequate intake of some nutrients

Food preferences in the diet have different effects on the environment and health [4]. The environmental footprint of foods varies across countries, where they are grown, and how they are grown [14]. Only a limited number of specific foods' environmental footprints (especially carbon footprints) have been assessed in a limited number of countries. This is a significant limitation in assessing the impacts of different diets in different countries [51]. The potential impacts of sustainable diets on the planet and human health are summarized in Fig. 2.

Springmann et al. evaluated the environmental burdens per kg of some foods. Generally, it has been determined that animal foods' carbon footprint, nitrogen, and phosphorus footprint (red meat, eggs, milk, etc.) are higher than plant foods. It has been determined that legumes' land and blue water footprint are higher than foods of animal origin, and wheat and rice's nitrogen and phosphorus footprint are higher than other foods, although not as much as foods of animal origin [52]. Furthermore, the authors emphasized that the footprint values found for nutrients are average and may vary regionally [52].

The environmental impacts of fruit and vegetable production can vary based on farming methods, such as greenhouse vs. open field, organic vs. traditional, and different climatic conditions [53]. For example, using organic agriculture instead of traditional agriculture in producing agricultural products can increase land use area and reduce pesticide residue [54].

High consumption of meat has adverse effects on the ecological systems of the planet [55]. The manufacture of animal-based food accounts for the majority of food-concerned GHG emissions because of low feed transformation efficiencies, fertilizer interested emissions, and enteric fermentation in ruminants, as well as 72–78% of total agricultural emissions; the feed-concerned effects of animal products account for about 10% of blue water use and 20–25% of cropland use, nitrogen, and phosphorus use [52]. Despite the adverse effects of consuming foods of animal origin on the environment, people continue to consume diets with high meat content [55]. In common, plant-based foods in the diet have a lesser environmental burden than animal-based food [52], and more resources are used to manufacture animal-based foods than plant-derived foods [56].

Although the environmental impact of plant protein source foods is lower than animal protein sources [52, 56], protein quality is an issue that should not be forgotten in terms of health. Proteins of plant and animal origin differ in terms of protein quality. Animal-derived proteins generally have all essential amino acids, while plant-based proteins lack one or more. Animal-based proteins also have better bioavailability than plant-based proteins [57].

An epidemiological study determined that declining meat consumption by 50% and altering it with fruits, vegetables, and grains could diminish GHG emissions by 19% and land use by 42% [58]. Another study determined that the production of foods with high animal protein content caused the highest contribution to land use and GHG emissions. In contrast, the production of vegetables and fruits contributed the most to water consumption [45]. Especially in high-income countries where meat and protein intake are consumed more than necessary, a trend towards a plant-based diet is thought to have favorable effects on both health and the environment [52].

In a study where integrated health and environmental modeling was carried out for more than 150 countries, it was determined that replacing 25–100% of animal-based foods with plant-based foods could reduce premature death rates, reduce greenhouse gas emissions by 20%−84% but increase freshwater use by 4%−16%, mainly in high-income countries. This modeling was less effective in countries where less animal products were consumed. However, it was determined that this transition was less effective in middle-income countries. It could reduce agricultural land, phosphorus, and nitrogen in middle-income and high-income countries but increase it in low-income and lower-middle-income countries [40]. In line with this study, the effectiveness of reducing the consumption of animal-derived foods on health and the environment depends on the level of animal-derived foods consumed in those countries. This situation suggests that in a country where small amounts of animal-based foods are consumed, reducing the consumption of these foods may have less impact on the environment and may also have adverse effects on health. Because foods of animal origin are a source of quality protein, rich in some vitamins and minerals, and have high protein bioavailability, this shows us the necessity of consuming these foods at sufficient levels [59].

Different food production methods also impact health, the environment, and productivity. Although the productivity of traditional agricultural methods is high, they have adverse effects on both health and the environment, such as loss of biodiversity, soil erosion and deterioration of soil health, eutrophication, adverse effects of pesticides on health, and increased greenhouse gas (GHG) emissions. Organic agriculture is considered an alternative to these adverse effects of conventional agriculture [60]. The positive effects of organic agriculture on the environment emerge over a long period. For example, it increases soil biodiversity and reduces nutrient losses, thus helping to maintain and increase soil fertility and reducing groundwater pollution because synthetic fertilizers and pesticides are not used [61]. Although it is not clear [62], it is thought that this farming method can reduce the greenhouse effect and global warming thanks to its ability to sequester carbon in the soil [61]. Studies have shown that organic agriculture can reduce nitrogen emissions and pesticide use [63] and increase social welfare and economic resilience [64]. However, it was also found that more land use may increase in organic farming than in conventional farming [63], and nutrient yields may be 20 percent lower [64].

Organic animal husbandry is based on respecting animal welfare and providing the most appropriate nutrition for animals [65]. The objectives of this production style include protecting the health of humans and animals and the environment and producing good quality products. Organic livestock farming offers an environmentally friendly approach to production thanks to its potential to reduce environmental pollution and nutrient losses in farm production [66]. In addition, organic animal husbandry enables food safety and sustainability to be together [67].

From a global perspective, plant-based nutrition instead of the consumption of animal-derived foods is becoming more and more popular every day in terms of protecting human and planetary health [68]. To this end, countries have launched campaigns to encourage meat-free days, such as 'Meatless Monday' and 'Veggie Thursday' [68].

### The Impact of a Planetary Health Diet on the Environment and Health

The EAT-Lancet Commission has recommended a reference diet [6], also named the "Planetary Health Diet" (PHD), to protect the health of humans and the planet [6, 69]. This diet is designed to optimize human health, reduce non-communicable diseases, and protect the planet by reducing the pressures of food systems on the environment [6]. The EAT-Lancet reference diet mainly stresses the consumption of whole grains, oil seeds, legumes, vegetables, fruits, and unsaturated fats, contains a low to modest consumption of poultry and seafood, and advocates that starchy vegetables, refined grains, red meat, processed meat, and added sugar should not be consumed at all or should be consumed sparingly [6]. Different indexes, such as the EAT-Lancet score [70] and WISH (World Index for Sustainability and Health) [71], have been improved to evaluate this reference diet. Cacau et al. [69] claimed that these developed indices were not sufficient and developed the "Planet Health Diet Index" (PHDI) for the reference diet. A high PHDI score is related to reduced CF and high diet quality [69].

In the Eat-Lancet reference diet, the recommended intake amounts for nutritional elements are not determined according to the latest dietary reference intakes recommended by the European Food Safety Authority (for Europe) or the Institute of Medicine (for the USA and Canada [72], the assumed iron bioavailability is not specified. It is assumed that zinc has a moderate level of bioavailability [6]. However, the amount of phytates in the diet is more than twice that used to describe minimum zinc bioavailability [73]. Furthermore, the tendency of the population globally towards insufficient physical activity [74], the energy content was calculated as 2500 kcal according to the state of being moderately active or highly active in addition. However, the requirements have been arranged according to gender and age [6]. Facts such as ignoring the requirements of women of reproductive age are the basis for concerns about whether this reference diet will meet their nutritional needs [72]. Accordingly, in a study in which micronutrient deficiencies of the PHD were evaluated, it was determined that the intake of vitamin B12, zinc, iron, and calcium was insufficient. Bell et al. suggested increasing animal-based foods and reducing phytate content in a planetary health diet to prevent these deficiencies [72]. In a study evaluating the data of 98,465 individuals in France, it has been determined that the increase in sticking to the EAT-Lancet reference diet resulted in a decrease in the prevalence of nutrient deficiencies, especially for vitamin B9 and vitamin C, excluding zinc and vitamin B12, which have high bioavailability [75].

A prospective cohort study of younger subjects (n = 298, age > 15 years old) found a higher grade of sticking to the EAT-Lancet diet and a negative correlation between dietary land use and GHG emissions [46]. Another study with a large sample found that higher adherence to a planetary health diet was associated with lower greenhouse gas emissions and higher water footprint and land use [47]. A study modeling a global transition to a planetary health diet evaluated 151 countries and predicted that greenhouse gas emissions could decrease in 101 countries. However, agricultural greenhouse gas emissions could increase by 12–283% in low- and middle-income countries. In other words, it has been determined that a global transition to this diet could reduce the per capita greenhouse gas emissions of about 60% of the world's population and increase them by 40%. Accordingly, it was emphasized that the country-specific impacts of the planetary health diet should be considered in climate change mitigation policy [42].

Studies evaluating the relationship between a planetary health diet and health have found that increased adherence to a planetary health diet may reduce the risk of diabetes [76], obesity [77], and cancer [78]. Adherence to this diet has been associated with lower levels of LDL, total cholesterol, and blood pressure, which are associated with CVD risk [79] and a lower risk of stroke [76]. However, some studies have found no association with CVD risk [78, 46]. Studies evaluating the relationship between sticking to this reference diet and cardiovascular disease risk elements have found different results in the literature [46, 78, 79]. In this context, more studies should be conducted on this relationship.

Although some studies have determined that the PHD may positively affect health [76–79], more studies are required to draw definitive conclusions. The positive influences of this diet on health may be associated with decreased consumption of refined grains, added sugar, processed/red meat, and increased consumption of fruits and vegetables. One point that should be noted in this reference diet is that there is a reduction in the intake of animal-derived nutrients and an increase in phytate intake. This condition may cause insufficient nutrient intake, such as vitamin B12, vitamin A, zinc, and iron, and may pose a risk for specific segments of society. Moreover, although the PHD was developed to reduce environmental impacts, studies have determined that it may affect the environment differently.

### The Impact of Plant-Based Diets on the Environment and Health

A plant-based diet includes diet patterns that emphasize plant foods such as fruits, vegetables, nuts and seeds, whole grains, and legumes and limit or suggest no consumption of animal foods [80]. In the literature, many different diets such as vegetarian, semi-vegetarian, Mediterranean diet (MD), Dietary Approaches to Stop Hypertension (DASH), planetary health diet (PHD), and Scandinavian diet can be involved in the concept of plant-based diet [80].

#### The Impact of Vegetarian/Vegan diets on the Environment and Health

Vegetarian diets are a subclass of plant-based diets that restrict all or part of the consumption of animal foods. According to their content, there are many different types of vegetarian diets [81]. People may choose a vegetarian diet for numerous reasons, such as their affection for animals, wanting to preserve the environment better, and treating and preventing diseases [82]. The transition to plant-based nutrition can potentially decrease nutrition-connected GHG emissions by 49%, land use by 76%, blue water use by 14%, green water use by 21%, and eutrophication by 49% [83]. The transition from omnivorous to ovo-lacto-vegetarian and vegan diets is related to rising environmental sustainability. The vegan diet causes approximately 50% lower GHG emissions than the omnivorous diet, and the ovo-lacto-vegetarian diet has approximately 30% lower GHG emissions [29]. For this reason, plant-based diets are becoming increasingly popular in maintaining planetary health [29, 83]. In a study evaluating the environmental impact of the diets of vegan, ovo-lacto vegetarian, and omnivorous individuals, it was determined that individuals with an omnivorous diet had a higher water footprint, ecological footprint, and carbon footprint than individuals who chose other diets and that there was no remarkable distinctness between Ovo-lacto vegetarian and vegan diets in terms of environmental pressure [38]. In addition, a study compared the effects of the MD and vegan diet on the environment and nutrition. It was stated that the MD has higher carbon dioxide emissions than the vegan diet, and this is due to the meat, fish, and dairy products it contains. It was also determined that the MD may cause more biodiversity loss (approximately 3 times) than the vegan diet. However, the study concluded that there were cases that were not evaluated in terms of nutritional quality. Therefore, it may be more positive in terms of sustainability to turn to a diet that combines the MD and vegan diet [37].

Besides the positive impacts of vegetarian diets on the environment, there are many positive effects on health. In systematic studies [84, 85], it has been found that individuals on vegetarian and vegan diets have better lipid profiles and glucose levels than omnivores. In addition, these diets have been found to reduce the risk of ischemic heart disease, diabetes, and cancer [84, 85]. However, it has also been determined that these diets may increase the risk of bone fractures [85].

The introduction of conventional plant-based diets (pescatarian, vegetarian, vegan diets) without professional supervision might increase the hazard of nutritional deficiencies in some segments of the population (the elderly, women of reproductive age, breastfeeding/ pregnant women, children/adolescents, and infants) [86]. Vegetarian diets can cause deficiencies in the intake of nutrients such as vitamin B12, vitamin D, zinc, iron, calcium, and omega-3 fatty acids [87]. Additionally, the plant/animal source ratio of this diet negatively impacts the bioavailability of nutrients such as zinc, iron, vitamin A, and essential amino acids. It may lead to inadequate intake of these nutrients [88]. Depending on these reasons, using dietary supplements or foods enriched with these nutrients in combination with these diets may protect against nutrient deficiency [87].

However, sustainable diets such as the MD, DASH, and New Nordic diet (NND), which are more acceptable than traditional plant-based diets, more flexible in consuming animal-derived foods, and can be diversified regionally, can compensate for the energy and nutritional requirements of diverse populations without the requirement for any nutritional education or nutritional supplements [86].

#### The Impact of Mediterranean Diet on the Environment and Health

The Mediterranean diet emphasizes plant-based foods (unrefined grains, legumes, fruits, and vegetables), olive oil as the primary fat source, low to moderate dairy (mainly yogurt and cheese), fish, poultry, eggs, and minimal red meat consumption. Wine is typically consumed in moderate amounts with meals [89].

In a study conducted with 525 adult individuals, higher levels of adherence to the MD and the EAT lancet diet were related to lower land use and GHG emissions and higher water use [45]. Although water use increases with adherence to these diets, researchers believe this does not have a negative impact because most fruits and vegetables are grown using treated wastewater, which reduces environmental pressure [45]. A cross-sectional study determined that the higher the adherence of older people to the energy-restricted MD, the lower the total dietary CO_2_ emissions, and it was concluded that the MD could protect the environment [48].

In review studies [90, 91], it has been determined that the MD has a lower carbon, water, nitrogen [90], and ecological footprint [91] than the Western diet. In addition, the MD was found to have similar dietary costs to other diets [91]. The MD has been found to have a lower carbon footprint and water footprint than the current Italian diet [36] and the New Nordic diet [44] but a higher than vegan dietary pattern [37].

Different modeling studies [41, 43] have found that shifting from a Western to a Mediterranean diet can reduce land use, greenhouse gas emissions, phosphorus [41, 43] and nitrogen use [41], and eutrophication due to phosphorus runoff [43]. Concerning water use, these studies give conflicting results [41, 43]. In a study conducted with a large sample (PREDIMED-Plus) in which individuals were encouraged to follow the MD for one year, an increase in adherence to the MD was determined at the end of the intervention and, with this increase in adherence, a decrease in diet-related GHG emissions, land use, and eutrophication was determined [49]. At the end of the 6-week sustainable nutrition education intervention conducted by Falakacılar et al. with 160 university students, it was determined that the students' diet quality and adherence to the MD increased, and the diets' carbon footprint and water footprint decreased [50]. These two intervention studies show that informing, encouraging, and educating the public about sustainable diets can positively affect health and the environment [49, 50].

In addition to these sustainable features of the MD, it has many positive effects on health. A comprehensive review evaluating both observational and intervention research on human health of the MD found that the MD provides a decline in the incidence of cardiovascular disease and risk factors such as diabetes, obesity, metabolic syndrome, hypertension, and dyslipidemia, which may increase the risk of cardiovascular disease. In addition, sticking to the MD has been found to decrease mortality, especially cardiovascular mortality, and therefore extend life expectancy [90]. A systematic review of seven prospective cohort studies found that greater adherence to the MD was remarkably related to a 9% reduction in the risk of overweight and/or obesity [92]. In different MD intervention studies, it was determined that total cholesterol [93], LDL cholesterol, glucose, triglyceride levels, BMI [94], and intrahepatic fat content decreased [95], butyrate synthesis-related gene expressions in microbiota and insulin sensitivity increased [93] in the intervention group compared to the control group. This shows that the MD has therapeutic as well as health-protective properties.

An updated meta-analysis found that the highest level of adherence to the MD was related to a lower risk of cancer-related death and a lower risk of head/neck cancer, stomach cancer, breast cancer, liver cancer, colorectal cancer, and prostate cancer [96]. In addition, it is thought that MD may positively affect chronic kidney disease due to its effect on lipid profile and blood pressure, endothelial function, and inflammation [97]. The 2020 Kidney Disease Outcomes Quality Initiative guidelines recommend that the MD improve lipid control in adults with chronic kidney disease stages 1–5, not on dialysis, and in transplant patients [98].

The MD has many advantages in terms of human health and planetary health. It has been recommended and continues to be recommended by many professionals since time immemorial. In particular, the transition from a Western-style diet to an MD is more straightforward than other more restrictive plant-based diets, and the MD is a reliable diet that is sustainable, does not carry any risk of nutrient deficiency, and can be applied at any age.

#### The Impact of the New Nordic Diet on the Environment and Health

The New Nordic Diet is characterized by the consumption of fruit and vegetables (cabbages, legumes, and root vegetables), potatoes, whole grains, nuts, locally grown herbs, fresh herbs, fish and shellfish, seaweed, mushrooms, small quantities of free-range livestock (inclusive poultry and pigs) and game [99]. It uses rapeseed/canola oil as the essential source of fat in the diet [100]. The basic guidelines of the diet are to consume less meat, more energy in plant foods, more fish and seafood, and more locally grown foods [101].

Very few studies in the literature evaluate the environmental impacts of NDD. The fact that this diet encourages local food consumption and emphasizes organic production makes a significant contribution to sustainability. A study found that MD and NND patterns have similar GHG emissions [39]. Another study evaluating the environmental impacts of the new Nordic diet pattern stated that this diet has a slightly higher carbon and water footprint than the MD. However, they are close so that the NDD could be an alternative to the MD [44]. The NND and the MD have a lot in common. Both diets are environmentally sustainable, emphasizing the consumption of locally and seasonally grown foods [100].

In large-sample studies [102, 103], higher adherence to the NND was associated with higher intake of fiber and higher intake of macro- and micronutrients, which positively affected diet quality; however, adherence was associated with increased consumption of red/processed meat [102, 103], potatoes and sweets [103]. Although reducing meat intake in the NND [101] is desirable, studies have found different results [102, 103].

In the literature, studies have shown that the Nordic diet has positive effects in improving cardiovascular health [104, 105], maintaining a healthy pregnancy [102], and reducing obesity-related cases (body fat ratio, body weight gain) [104, 106]. In addition, the NND was modified to reduce phosphorus, sodium, and protein intake, and the New Nordic Renal Diet (NNRD) was developed specifically for chronic kidney disease patients [107]. In a 26-week randomized controlled trial conducted in mildly overweight individuals with stage 3–4 chronic kidney disease, it was determined that urinary phosphorus and protein excretion decreased in the New Nordic Renal diet group compared to the control group, and there was a substantial reduction in systolic blood pressure and body weight [108]. Although it has been determined that MD positively impacts chronic disease prevention, there are no definite results regarding the impacts of the Nordic diet on health. In this context, more research should be performed to confirm the potential positive influences of the Nordic diet on health [100].

The principles of the NND in terms of both human health and environmental sustainability suggest that it may be a potential diet for planetary health. However, in some studies, it has been determined that adherence to this diet may increase the consumption of foods such as meat [102, 103] and potatoes [103]. Therefore, more studies are needed to assess the effects of NND on human health and the environment.

#### The Impact of DASH (Dietary Approaches to Stop Hypertension) Diet on the Environment and Health

The DASH diet aims to reduce blood pressure by consuming foods low in sodium, cholesterol, saturated fat, and total fat content and high in fiber, protein, calcium, magnesium, and potassium content [109]. The diet emphasizes the consumption of high amounts of vegetables and fruits, protein, whole grains, fiber, lean or low-fat dairy products, oilseeds, fish, poultry, and [110] while limiting the consumption of foods high in saturated fat, such as fatty meats and full-fat dairy products, tropical oils (palm oil, coconut, and palm kernels), sugar-sweetened beverages, refined grains, and sweets [111].

The number of studies examining the environmental footprints of adherence to the DASH diet is very few in the literature. One study determined that higher adherence to the DASH diet is related to lower GHG emissions but may lead to higher costs [112]. A study by Kling et al. found that diets with the highest adherence to the DASH diet resulted in 25–50% lower greenhouse gas emissions than those with the lowest adherence, as well as reduced land use caused by the protein foods (both animal and plant) included in the diet [113].

It has been determined that the DASH diet can positively impact health [109]. In a meta-analysis, it was found that the DASH diet remarkably improved cardiovascular risk factors (LDL, total cholesterol, diastolic blood pressure, systolic blood pressure), and as a result of this improvement, it could provide about 13% reduction in the 10-year Framingham risk score [114]. It has been determined that the DASH diet has positive impacts on reducing blood pressure in individuals without and with hypertension, and even the blood pressure-reducing influence of the DASH diet is higher in younger individuals with high daily sodium consumption [115]. In addition, it has been found that the DASH diet can positively affect insulin sensitivity independent of body weight [116] and potentially reduce body weight [117]. A case–control study discovered that sticking to the DASH diet can decrease the risk of breast cancer by about 30% [118]. Increased adherence to the adjusted DASH diet has been related to a lower risk of breast [118, 119] and stomach cancer [120]. When cross-sectional studies in a meta-analysis were evaluated, it was determined that an increase in sticking to the DASH diet lessened the risk of kidney disease by 32% [121]. Although the DASH diet positively affects health, its effects on the environment need to be evaluated with more studies.

## Conclusion

One of the leading human activities that pushes planetary boundaries is food production. The damage done to the planet while food is being produced could be one of the primary reasons why we will not have enough food in the future. Human dietary patterns influence the environment by driving food systems and are among the most important determinants of human health. However, no consensus exists on which diet is best for planetary and human health. Planetary health diets, plant-based diets, and vegetarian/vegan diets restrict the consumption of animal foods and products and encourage the consumption of plant-based foods; thus, these diets are thought to reduce environmental pressure. However, it should not be forgotten that these diets cannot have the same effect in every country and may have different effects depending on the nutritional culture of the countries (for example, having low meat consumption in normal conditions), development level, income level, differences in food systems (organic farming practice, intensified farming, waste management, etc.). In particular, differences in food production systems across countries can change the effectiveness of foods on the environment and health. Sustainable transformation of food systems is essential for sustainable diets to realize their potential positive environmental impact. In this context, when providing nutritional advice regarding sustainability, it is important to include details about food production, such as "reducing traditionally produced red meat and animal-derived products."

Furthermore, diets have mainly been evaluated for their carbon, water, and land footprints. More studies evaluating different footprints are needed. Countries determine the environmental footprint loads of the foods produced in their countries depending on their food systems, and using these footprint data in their studies may provide more accurate results in evaluating environmental impact.

Diets such as traditional plant-based diets (vegan, etc.) and planetary health diets can lead to nutrient deficiencies if they are not implemented with the support of a professional or if there is insufficient nutritional knowledge. These diets may cause deficiencies in nutrients that can only be obtained from animal-based foods or whose bioavailability is higher in animal-based foods, such as B12, zinc, iron, calcium, and essential amino acids. It can pose a risk, especially for infants, children/adolescents, women, pregnant/breastfeeding women, and the elderly. Sustainable diets such as the MD, the New Nordic diet, and the DASH diet are more flexible and more acceptable to all segments of society. Even so, further studies are needed to confirm the environmental effects of both the New Nordic and DASH diets. However, the impact of the Mediterranean diet on the environment and health has been more comprehensively evaluated, and this diet is more prominent than other diets in terms of environmental and health protection. Nevertheless, due to differences in countries and food systems, it is difficult to recommend a standard diet for the whole world that protects the environment and health. Instead of a globally recommended reference diet to protect the planet and human health, each country's analysis of its food systems, use of the most appropriate sustainable food production methods, choosing a sustainable diet style, and creating policies accordingly can help achieve sustainable goals more quickly.

## Data Availability

No datasets were generated or analysed during the current study.
